# News and Coming Events

**Published:** 1907-08-10

**Authors:** 


### NEWS AND COMING EVENTS.

Mr. Andrew Carnegie has contributed a sum of £100,000 to the King Edward Hospital Fund for London. The gift is made unconditionally, but in a covering letter the donor expresses a desire that it shall be used as the administrators of the Fund may deem best "the more strenuously for reform the better."

---

The Nettleship Medal, presented by the Ophthalmological Society, has been awarded to Mr. Herbert Parsons for his monograph on the "Pathology of the Eye."

---

Lady Ludlow has received a donation of one hundred guineas from the Mercers' Company in aid of the fund for the new Nurses' Home in connection with Bartholomew's Hospital.

---

A vacation course will be held at the North-Eastern London Post Graduate College, Prince of Wales's Hospital, Tottenham, N., commencing on September 9. Full particulars may be obtained on application to the Dean at the hospital.

---

The first meeting of the medical section of the Royal Society of Medicine will be held on October 22nd next. Dr. Hector Mackenzie will open a discussion on the complications and sequelæ of pneumonia and the treatment of pneumococcal infections by serums or vaccines.

---

Founder's Day at Epsom College was commemorated on Saturday, July 27th, when a handsome jubilee memorial window was unveiled in the college chapel. The window, which has cost £700, is the gift of old Epsomians and friends of the college, and is from designs of Mr. James Powell.

---

The International Congress of Psychiatry and Neurology will hold its meetings in Amsterdam this year. The meetings will be held from the 2nd to the 7th of next month (September), and an interesting agenda has been drawn up. The programme of excursions, visits to asylums, and social functions is a long one, and the meeting is expected to be fully representative.

---

The second International Congress on School Hygiene was opened at the University of London on August 5 by the Earl of Crewe. The inaugural address was delivered by Sir Lauder Brunton, LL.D., M.D., D.Sc., F.R.C.P., F.R.S., the President of the Congress, who, in the course of his address, spoke of the complexity of conditions in a civilised community which made the natural desire which we all feel that our children should grow up healthy and well-educated less simple than it is in a savage state, where the chief objects of life are war and hunting. Under existing conditions, education has a tendency to cultivate one or two faculties of the mind, especially memory, to the injury of others, while till lately the condition of the body, as servant of the mind, has been lost sight of in this country. This is now being repaired, and an important part of school hygiene is the medical inspection of school children. It is important to learn the defects of eyes, ears, nose, and teeth which may interfere with the progress of individual scholars, and to know of those hidden weaknesses of heart and lungs which may render the physical exercises, suitable for others, dangerous to a child with a weak heart, and make a child suffering from phthisis unsuitable for school life, and a source of danger to his companions. By the early detection and isolation of infected children the necessity for closing schools may be, to a great extent, prevented, the spread of skin diseases completely checked, and even a fatal disease like diphtheria may be almost stamped out. Sir Lauder spoke of the recent developments of education in the matter of teaching the mentally deficient according to their capacity, and giving training in handicrafts to cripples, but pointed out that with regard to the latter, medical inspection may in many cases detect the tuberculosis of the joints that causes most of the distorted limbs, and with proper treatment prevent their becoming cripples. He spoke highly of the influence of rifle clubs. Shooting with rifles properly adapted to their size and strength is an exercise which tends to bring out many of the best qualities of boys, and has even a moral effect on those belonging to them, who regard a son's success in shooting as an honour which it behoves them to live up to. Moreover, a love for shooting prevents boys becoming intemperate, because they know that intemperance makes their hands unsteady, and spoils their chances. In conclusion, Sir Lauder Brunton pointed out the necessity of schoolmasters and schoolmistresses working hand in hand with doctors in all attempts to ensure the physical welfare of school children, and advised that instead of spending money in pensioning old people, we should expend the money on our children, so that in their active lives they can provide pensions for themselves. At the conclusion of the address the thanks of the meeting to the speaker were moved by Professor Griesbach (Germany), seconded by Dr. L. H. Gulick (U.S.A.), and supported by Sir Alexander Pedlar (India). A vote of thanks to Lord Crewe was proposed by Dr. Mathieu (Paris), seconded by Sir Philip Jones (New South Wales), and supported by Dr. Leo Burgerstein (Austria).

---

Infant Consultations.—In the early part of 1906 the Directors of the St. Marylebone General Dispensary gave permission to the medical staff of that charity to co-operate with the members of the local Health Society in organising a systematic campaign to deal with the preventible causes of infantile mortality in the district. The result was the inauguration of a system of "Infant Consultations" similar to that of the Charité in Paris. The first report of the work done has just been issued, and proves well how effective such a system can be made to be under proper and efficient management. A total of 98 babies were brought to these consultations during the past year—a small but encouraging number when it is borne in mind that Professor Budin, when he started his consultations at Clinique Tarnier, and as an inducement for mothers to bring their children offered a free distribution of sterilised milk, had only 78 attendances during his first year. The majority of these cases were "difficult," but only two deaths were recorded, and satisfactory progress was made in 76 per cent. of cases. It is interesting to note how the mothers look upon the new scheme. In general it appears that the system is viewed with approval by Marylebone motherhood. One lady, however, "discontinued her visits because she did not like the defects of her child pointed out; another because she was told that two of her older children were rickety, and that she must alter her method of bringing up her youngest; two others because no medicine was supplied; and a fifth because she objected to her baby being weighed on the same scale with other babies." A meeting will be held at the Portman Rooms, Dorset Street, on Tuesday, July 2, at 4 p.m. to receive the first year's report. Lord Robert Cecil will occupy the chair, and the prizes and certificates will be distributed by the Duchess of Sutherland.

